# Clinical implications of the biomechanics of bicuspid aortic valve and bicuspid aortopathy

**DOI:** 10.3389/fcvm.2022.922353

**Published:** 2022-08-12

**Authors:** Ali Fatehi Hassanabad, Melissa A. King, Elena Di Martino, Paul W. M. Fedak, Julio Garcia

**Affiliations:** ^1^Section of Cardiac Surgery, Department of Cardiac Sciences, Cumming School of Medicine, Libin Cardiovascular Institute, Calgary, AB, Canada; ^2^Department of Civil Engineering, University of Calgary, Calgary, AB, Canada; ^3^Libin Cardiovascular Institute, University of Calgary, Calgary, AB, Canada; ^4^Centre for Bioengineering Research and Education, University of Calgary, Calgary, AB, Canada; ^5^Department of Cardiac Sciences, Cumming School of Medicine, University of Calgary, Calgary, AB, Canada; ^6^Stephenson Cardiac Imaging Centre, Libin Cardiovascular Institute, Calgary, AB, Canada; ^7^Department of Radiology, University of Calgary, Calgary, AB, Canada; ^8^Alberta Children's Hospital Research Institute, University of Calgary, Calgary, AB, Canada

**Keywords:** bicuspid aortic valve, bicuspid aortopathy, biomechanics, BAV-mediated hemodynamics, 4D flow MRI

## Abstract

Bicuspid aortic valve (BAV), which affects up to 2% of the general population, results from the abnormal fusion of the cusps of the aortic valve. Patients with BAV are at a higher risk for developing aortic dilatation, a condition known as bicuspid aortopathy, which is associated with potentially life-threatening sequelae such as aortic dissection and aortic rupture. Although BAV biomechanics have been shown to contribute to aortopathy, their precise impact is yet to be delineated. Herein, we present the latest literature related to BAV biomechanics. We present the most recent definitions and classifications for BAV. We also summarize the current evidence pertaining to the mechanisms that drive bicuspid aortopathy. We highlight how aberrant flow patterns can contribute to the development of aortic dilatation. Finally, we discuss the role cardiac magnetic resonance imaging can have in assessing and managing patient with BAV and bicuspid aortopathy.

## Introduction

Bicuspid aortic valve (BAV) is the most common type of congenital heart disease (CHD), affecting 0.5–2% of the general population (1–3). BAV results from the fusion of the cusps of the aortic valve, which is normally a tri-leaflet valve that facilitates the flow of oxygenated blood from the left ventricle into the aorta. Different fusion patterns of the three leaflets have been identified to lead to a bicuspid morphology. Various classifications have also been proposed to describe BAV, including Sievers, Schaefer, and Michelena (4–6). The pathophysiology of BAV is not well understood, but genetics are thought to have a role (7–9). Nevertheless, different fusion patterns of BAV have been shown to result in aberrant blood flow dynamics through the aortic valve (10). This dysregulation in the blood flow can contribute to dilatation of the aorta, a condition known as bicuspid aortopathy (10). Bicuspid aortopathy can predispose patients to aortic dissection, which is associated with high rates of morbidity and mortality (11). Given these important clinical implications, various groups have aimed to better understand the pathophysiology of BAV and bicuspid aortopathy. Cardiovascular societies have also suggested standardizing the definition and classification for BAV. Moreover, biomedical engineers are striving to elucidate the biomechanics of BAV and bicuspid aortopathy. Their findings, coupled with innovative multi-modality imaging techniques, have tremendously helped improve our knowledge of the natural history of BAV and bicuspid aortopathy. Herein, we review the latest literature on BAV and bicuspid aortopathy as it pertains to clinical concepts, biomechanics, and non-invasive imaging. We also summarize the literature pertaining to BAV and bicuspid aortopathy biomechanics. Finally, we explore whether multimodality imaging techniques can be used to better inform clinical decision-making algorithms for patients with a BAV.

## Epidemiology

Obtaining an accurate estimate of the incidence of BAV in the adult population is not trivial. Most data stems from autopsy series. In the largest autopsy series of 21,417 consecutive autopsies, Larson and colleagues identified 293 (1.37%) patients with BAV (12). Pauperico et al. studied 2,000 cadaveric aortic valves and found only 13 (0.65%) of them to be bicuspid (13). Roberts (14) and Datta (15) also used autopsy series to determine the prevalence of BAV. Three studies have also aimed to determine the prevalence of BAV in alive patients (16–18). Basso et al. screened 817 primary school children using transthoracic echocardiography (TTE) and found 0.5% of the population to have BAV, where there was a higher incidence in males when compared to females (0.75 vs. 0.24%) (16). Tutar and colleagues studied 1,075 newborns to determine the prevalence of BAV in neonates (17). They also used TTE and found BAV to be present in 0.46% of live births, where 0.71% of male neonates had BAV compared to 0.19% of females (17). Sillesen et al. performed a cross-sectional, population-based study on all newborns born in Copenhagen (18). In total, 25,556 underwent TTE and BAV was found in 196 newborns, where they noted a 2:1 ratio for males to females (18). Other studies have also demonstrated BAV to be more common in males, suggesting a potential genetic predisposition for this CHD (9, 19). With respect to non-syndromic BAV, it has been found to be associated with multifactorial inheritance, low penetrance, and variable phenotypes (20). It has been postulated that reduced number of X chromosome genes that evade inactivation could explain the higher frequency of BAV in men (20). Various studies have also shown BAV to be common in XO Turner syndrome where more than 30% of patients with Turner syndrome have BAV (1, 21). The relative prevalence of BAV in men and women is important since different studies have found that clinical outcomes can be heavily influenced by sex (19, 22–24).

## Pathophysiology

There is strong evidence supporting an underlying genetic predisposition for acquiring BAV, where it has been found to cluster in families (25–27). Variance component methodology and modeling have established the heritability of BAV to be up to 89%, suggesting an almost exclusive genetic cause (8, 28–30). There is also mounting clinical data from familial studies that have shown BAV to have an autosomal dominant pattern of inheritance with reduced penetrance and variable expressivity (31). Moreover, BAV is associated with aortic coarctation, patent ductus arteriosus, and anomalies in proximal coronary artery anatomy (9, 32). Nevertheless, the specific genes and genetic abnormalities that result in BAV are yet to be defined. Indeed, a host of genes, including *NOTCH 1, FBN1*, and *TGF*β*1/2*, with divergent inheritance pattern can also contribute to the development of BAV (31, 33–39). *NOTCH1* is a transmembrane receptor that plays a role in valvulogenesis and extracellular matrix (ECM) modeling, and its loss of function leads to the development of BAV (40). However, mutations in *NOTCH1* account for <5% of BAV cases, with most occurring sporadically with no known heritable thoracic aortic aneurysm gene identified (6).

Moreover, as noted above, although BAV is usually present in isolation, it is linked with other genetic syndromes, such as Andersen syndrome, Turner syndrome, William Beuren, Bosley-Salih-Alorainy, Tetralogy of Fallot, hypoplastic left heart syndrome, and Athabascan Brainstem Dysgenesis syndrome (33, 41). BAV may also be present in patients with connective tissue diseases including familial Type A aortic dissection, Marfan syndrome, Loeys-Dietz syndrome, and vascular Ehlers-Danlos syndrome (41). Indeed, there is evidence showing that, in patients with Marfan syndrome, the prevalence of BAV was more than four times higher than what has been shown in the general population (16, 42, 43). Table 1, which is adapted from Giusti and colleagues (44) summarizes the genes that have been implicated in the BAV patient population. Germane to this manuscript, there is also literature supporting the notion that BAV can be non-syndromic, where functional and hemodynamic factors can modulate valve phenotype and morphology during development leading to either a tricuspid aortic valve or a BAV and its various types, respectively (45–48).

**Table 1:** Summary of the genes that have been implicated in the BAV patient population [adapted from Giusti et al. (44)].

**Genetic loci**	**Function or associated syndrome**
*NOTCH1*	Embryonic valve maturation and regulation of aortic valve calcification
*GATA5*	Mediates cell differentiation during embryonic cardio genesis
*GATA4*	Involved in cardiac embryogenesis
*ACTA2*	Smooth muscle α-actin
*UFD1L*	Development of ectoderm-derived structures during embryogenesis
*AXIN1/PDIA2*	Regulates valvulogenesis and cardiac neural crest development through TGF-β signaling (PDIA2 role unknown)
*ENG*	Important in valvulogenesis
*FBN1*	Marfan syndrome
*KCNJ2*	Andersen syndrome
45 XO karyotype	Turner syndrome
Deletion of 7q11.3 including *CLIP2, ELN, GTF2I, GTF2IRD1*, and *LIMK1*	William Beuren syndrome
*HOXA1*	Bosley-Salih-Alorainy syndrome, Athabascan brainstem dysgenesis syndrome
*TGFBR1/2*	Loeys-Dietz syndrome
*COL3A1*	Vascular Ehler Danlos syndrome
*ACTA2*	Thoracic aortic aneurysm and dissection syndrome

## Classifications and nomenclature

**Figure 1:**
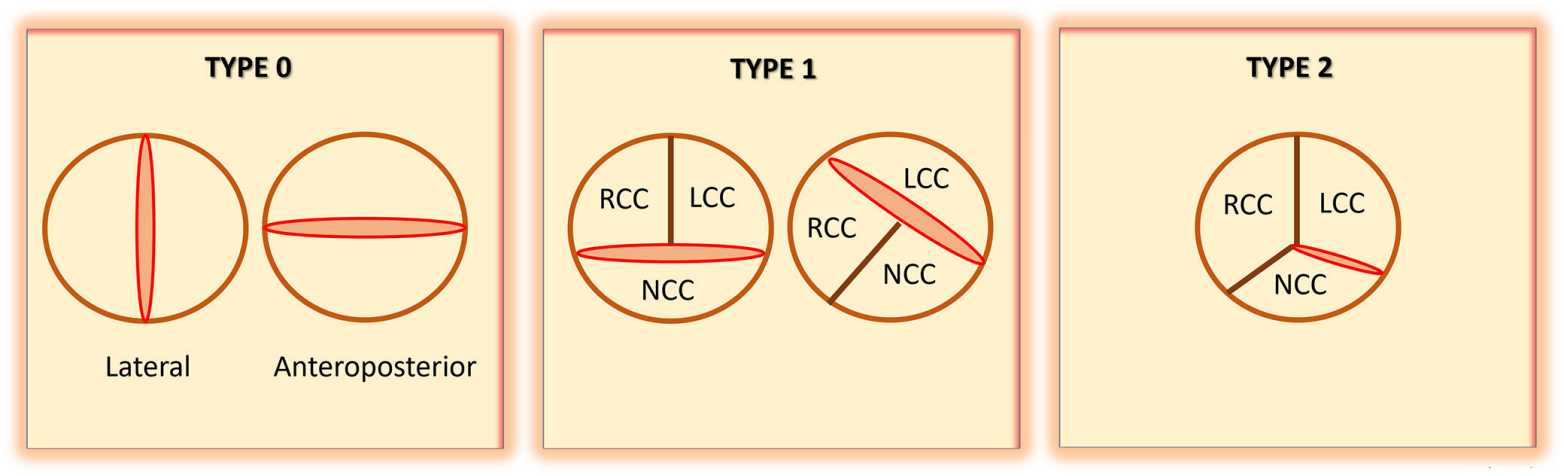
Schematic representation of bicuspid aortic valve (BAV), as defined by Sievers and Schmidtke [modified from Sievers et al. (4)]. RCC, right coronary cusp; LCC, left coronary cusp; NCC, non-coronary cusp.

One of the challenges with BAV literature and research has been the heterogeneity of definitions and classifications that have been used. Further compounding this complexity, assigned nomenclature has been based on pathology specimens and images obtained from echocardiography, CT scans, and cardiac magnetic resonance (CMR) imaging. Since the 1970's, 11 published definitions have been proposed to describe BAV fusion patterns (49). Using pathology specimens, Roberts was the first to employ “anterior-posterior” and “right-left” terminology (14). Bradenburg used echocardiography images and a clock-face nomenclature (50), while Angelini used autopsy specimens and based the classification on the presence of a raphe and employed anterior-posterior/right-left cusps terminology (51). Sabet et al. also used the presence or absence of raphe on 534 cadaveric specimens to define BAV (52). Probably the most commonly used nomenclature and classification for BAV is the one suggested by Sievers and Schmidtke (4) (Figure 1). Using autopsy specimens from 304 cadavers, they established the “type” of BAV based on the presence of raphe: Type 0: no raphe; type 1: 1 raphe; and type 2: 2 raphes. Other definitions and classifications have been proposed by Schaefer (5), Kang (53), Michelena (6), Jilaihawi (54), Sun (55), and Murphy (56). Table 2 summarizes these definitions and classifications. The diverse heterogeneity in BAV nomenclature has been confusing. Some of the proposed categories for BAV have also had limited clinical application. To address these challenges, an international consensus statement on BAV nomenclature and classification was prepared and published in 2021 (49). This statement, which has been endorsed by major cardiovascular societies, recognizes three types of bicuspid valves. First, the fused type that has either a right-left cusp fusion, or right-non-coronary cusp fusion, or left-non-coronary cusp fusion phenotype. Second, the 2-sinus type, which has latero-lateral and antero-posterior phenotypes. Third, is the partial-fusion type. Based on this categorization, BAV right to left cusp fusion (R-L) is the most prevalent (70-80%), followed by right to non-coronary cusp fusion (R-N) (20–30%), and least commonly, left to non-coronary cusp fusion (L-N) (3–6%). The consensus statement also emphasizes that the presence of raphe and the symmetry of the fused type phenotypes are critical aspects to describe. Using a standardized nomenclature should help in simplifying BAV literature. Clinical application and correlation of the various types of BAV can also be better conveyed.

**Table 2:** A summary of the definitions and classifications that have been used in the BAV literature [adapted from Michelena et al. (49)].

**References**	**Type of study**	**Nomenclature**
Roberts (14)	Pathology	Anterior-posterior cusps, right-left cusps, presence of raphe
Brandenburg et al. (50)	Echocardiography	Clock-face nomenclature: Commissures at 4–10 o'clock with raphe at 2 o'clock (R-L) Commissures at 1–6 o'clock with raphe at 10 o'clock (R-N) Commissures at 3–9 o'clock without raphe (L-N)
Angelini et al. (51)	Pathology	Anterior-posterior cusps, right-left cusps, presence of raphe
Sabet et al. (52)	Pathology	RL, RN, LN, presence of raphe
Sievers and Schmidtke (4)	Pathology	Type 0 (no raphe): anteroposterior or lateral cusps (true BAV) Type 1 (1 raphe): R-L, RN, L-N Type 2 (2 raphes): L-R, RN
Schaefer et al. (5)	Echocardiography	Type 1: RL
		Type 2: RN
		Type 3: LN
		Presence of raphe
		Aorta: Type N: normal shape Type E: sinus effacement Type A: ascending aorta dilatatio
Kang et al. (53)	Computed tomography	Anteroposterior orientation: type 1: R-L with raphe type; 2: R-L without raphe
		Right–left orientation: Type 3: RN with raphe Type 4: L-N with raphe Type 5: symmetrical cusps with 1 coronary artery originating from each cusp
		Aorta: Type 0: normal Type 1: dilated root Type 2: dilated ascending aorta Type 3: diffuse involvement of the ascending aorta and arch
Michelena et al. (6)	Echocardiography	Type 1: R-L Type 2: RN Type 3: L-N Presence of raphe
Jilaibawi et al. (54)	Computed tomography	Tricommissural: functional or acquired bicuspidity of a trileaflet valve
		Bicommissural with raphe Bicommissural without raphe
Sun et al. (55)	Echocardiography	Dichotomous nomenclature: R-L Mixed: (RN or L-N)
Murphy et al. (56)	Cardiac resonance imaging	Clock-face nomenclature: Type 0: partial fusion/eccentric leaflet Type 1: RN, RL, LN partial fusion/eccentric leaflet Type 2: RL and RN, RL and LN, RN and LN partial fusion/ eccentric leaflet

## Clinical significance of BAV and bicuspid aortopathy

The clinical implications of BAV and bicuspid aortopathy are noteworthy. BAV is associated with aortic valve insufficiency (AI), aortic valve stenosis (AS), infective endocarditis, aortic dilatation, and aortic dissection (48). While AI is present in approximately 30% of BAV patients, AS is much more common. Furthermore, over half of patients with BAV develop aortic dilatation and this may progress to aneurysm, dissection, and rupture, which are frequently lethal complications (49). Although BAV can cause morbidity and mortality through valvular disease and bicuspid aortopathy, the overall survival for patients with a BAV is similar to that of the non-BAV population (57). Thus, if surgical intervention is provided at the appropriate time, BAV patients should benefit from identical survival rates to non-BAV patients. Salient to clinical management, bicuspid aortopathy can have varied presentations, where dilatation may occur in the aortic root, the ascending aorta, the proximal aortic arch, or any combination of these sites (58). The tubular ascending aorta is the most common segment affected in bicuspid aortopathy, where 60–70% of aneurysms occur at this site (59). When aortopathy occurs at the level of the aortic root, the sinuses of Valsalva are predominantly affected (59). There is evidence suggesting that dilatation of this segment is associated with more rapidly progressive aortopathy (60–62). In contrast, non-BAV aortic aneurysms, such as degenerative aneurysms, tend to begin in the mid ascending aorta and progress distally and proximally, while aneurysms secondary to connective tissue diseases are limited to the aortic root (59).

The clinical manifestation of BAV-associated complications is heterogenous, spanning from some patients developing deadly aortic dilatation and subsequent dissection, and others suffering no symptoms at all (49). Various studies have quantified the risk over time of developing an aneurysm in the ascending aorta in BAV patients. These studies have found that 20–30% of BAV patients have aortic dilatation during a follow-up of 9–25 years (6, 57, 63). Critically, studies have found that the risk of acquiring aortic dilatation was 80 times higher in BAV patients compared to the general population (6). Furthermore, recent studies have found that the rate of aortic dissection in patients with BAV 15 years after aortic valve replacement (AVR) was 0.55%, which is not significantly higher than that for patients with tricuspid aortic valve (0.41%) (63–65). Studies have also aimed at better understanding the difference between aortic aneurysms in patients with and without a BAV. Patients with BAV presented at a smaller aortic diameter; their aortic aneurysms grew more rapidly; and a higher number of BAV patients required surgical treatment at a significantly younger age compared to non-BAV patients with an aortic aneurysm (66). The same study found that BAV patients who had aortic valve stenosis and aortic dilatation were at an increased risk for aortic dissection, rupture, or death before surgery compared to patients with normally functioning BAV (66). It is also important to emphasize that the absence of valvular dysfunction does not lessen the risk of aortic dissection in BAV patients (67). Conversely, BAV patients who also have aortic stenosis or aortic regurgitation are at an increased risk of aortic dissection and rupture (68). Interestingly, BAV patients who also have aortic regurgitation are more prone to aortic dissection (69).

It is important to summarize the contemporary clinical outcomes for patients with BAV, which have been extensively covered by Michelena and colleagues from the International BAV Consortium (6). Excellent overall survival rates have been found for patients with BAV in community, population-based studies, while outcomes are poorer in referral center patients who have required aortic valve replacement. Heart failure is uncommon in patients with BAV, and aortic stenosis is a more common indication for surgery compared to aortic insufficiency. Development of aortic aneurysm (aortic diameter >45 mm) occurs in 25–45% of patients over prolonged periods of follow-up, but aortic dissection is a rare event (~1%) outside of tertiary referral center populations, where it is more common (~10%) (6).

Collectively, the diversity in BAV and bicuspid aortopathy complicates management as interventions must coincide with risk of developing aortic complications, which is challenging to determine in a clinically heterogenous population. Ideally, non-invasive biomarkers that have high prognostic sensitivity and specificity should be established to accurately predict the natural course of BAV-associated complications (70). Such biomarkers, which do not require invasive approaches to obtain them, can then be used to intervene on the appropriate patient at the most optimal time to prevent serious complications (71–73).

## Bicuspid aortopathy: Genetics or valve-mediated dysfunctional flow patterns and aortic wall shear stress?

BAV is associated with an increase in aortic wall shear stress (WSS) (74–76), the tangential shear force that blood flow exerts on a vessel wall, and thus potentially expression of aortopathy. Four-dimensional flow magnetic resonance imaging (4-D flow MRI) can be used to assess for WSS, the aortic valve, and the thoracic aorta (77). Utilizing novel imaging techniques such as 4D flow MRI presents an opportunity to characterize and risk stratify patients with BAV who are at risk of severe aortic complications (78). The mechanism underlying bicuspid aortopathy is a widely debated subject, oscillating between genetic predisposition to hemodynamic causes. Like BAV, *NOTCH 1* is the gene that has been implicated in bicuspid aortopathy (48). A study of first-degree relatives of BAV patients with tricuspid valves (TAV) were found to have altered aortic shape and hemodynamics despite the absence of valvular disease or aortic dilatation. These findings suggest the presence of an unidentified genetic component of BAV aortopathy that is independent of bicuspid valve pathology (79). Bicuspid aortopathy frequently affects the ascending aorta and rarely the descending aorta, and these regions have distinct embryological origins (30). Smooth muscle cells derived from the ascending aorta of BAV patients also demonstrate impaired contractility, further suggesting that bicuspid aortopathy may develop from a genetic defect that results in abnormal differentiation (80).

**Figure 2:**
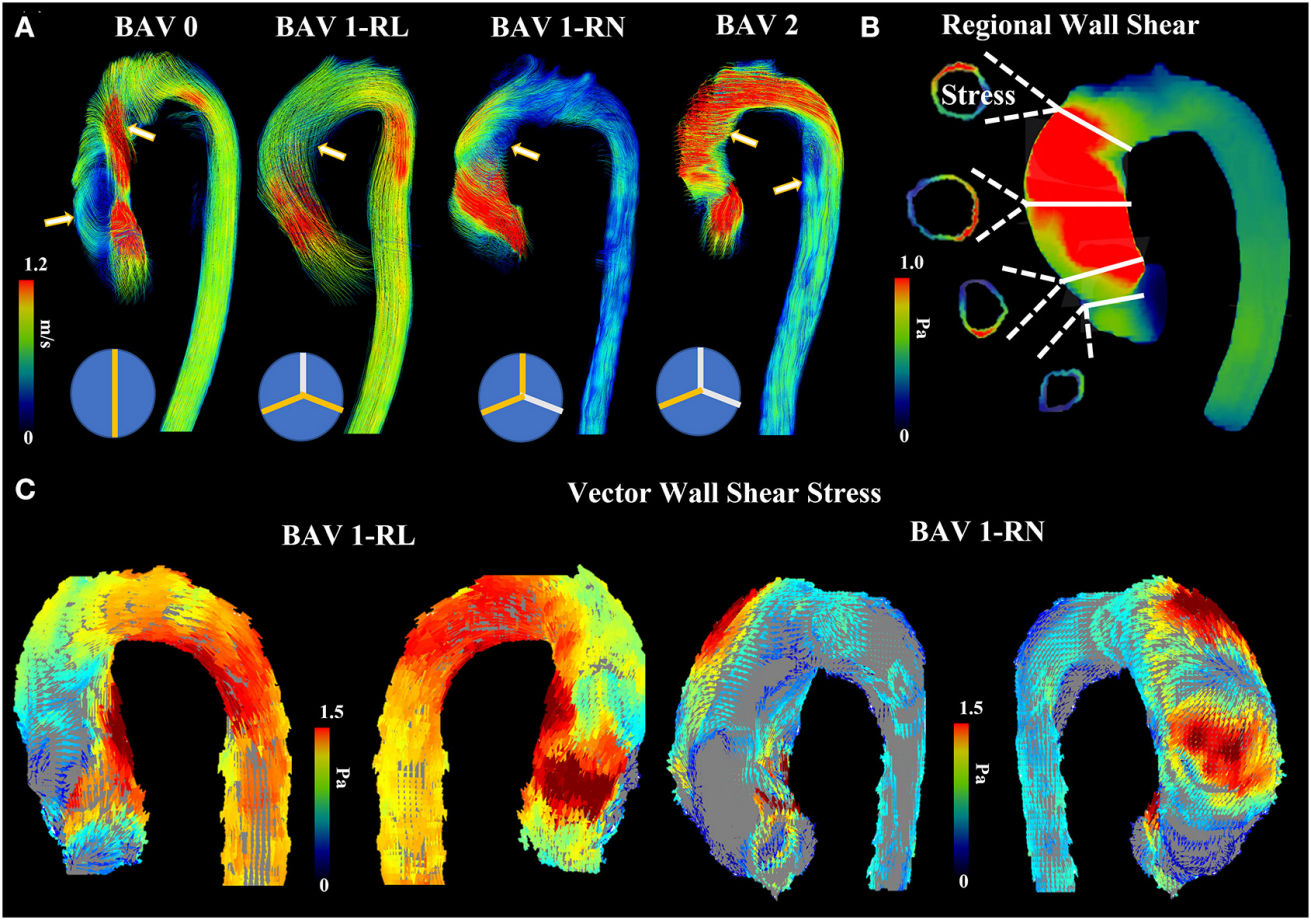
Abnormal bicuspid aortic valve (BAV) hemodynamics. **(A)** shows four patients with different BAV phenotype. Arrows point to regions where helical and abnormal flow patterns can be observed. **(B)** shows an example of regional wall shear stress in the ascending aorta. Four landmark locations are illustrated: left ventricular outflow tract, sinus of Valsalva, mid-ascending aorta, and distal ascending aorta. **(C)** shows anterior and posterior view from vectorial wall shear stress in RL and RN patients.

In contrast, the hemodynamic theory of bicuspid aortopathy postulates that BAV cusp fusion leads to subclinical stenosis resulting in abnormal hemodynamics and increased WSS, ultimately eliciting aortic remodeling (81). In a tricuspid aortic valve (TAV) the systolic velocity jet is unidirectional and does not abnormally impinge on the aortic wall. The abnormal hemodynamics observed in BAV are determined by the phenotype of valve cusp fusion (Figure 2A). The fusion pattern of the cusps and their opening angle, which is a quantifier of the restricted cusp motion, influences aortic root shape eccentricity, with a larger sinus located at the non-fused cusp (82), and aortic aneurysm growth rate, respectively (47). Alterations in ventricular outflow hemodynamics leads to varying levels of WSS at certain regions of the aorta as detected by 4D flow MRI (Figure 2B). For example, A R-L BAV velocity jet is directed anteriorly with greater axial WSS at the aortic root, while a R-N BAV jet is directed toward the posterior aorta with greater circumferential WSS at the mid-distal aorta (30, 83) (Figure 2C).

## Clinical implications of bicuspid aortopathy

These conflicting theories have made management strategies difficult, where practices vary among cardiac surgeons with respect to the timing of intervention and the extent of a reparative operation (84). In BAV patients with a higher genetic predisposition to aortopathy, such as those with *NOTCH1* mutations, prophylactic surgical resection of the aorta may be warranted to prevent serious complications. However, in cases of BAV where aortopathy is presumed to be secondary to altered biomechanics and blood flow dynamics to a BAV, conservative management and/or aortic valve replacement (AVR) may be the preferred approach (85). Strategies to identify which patients would benefit from surgery is critical since an operation is not without risks. Ascending aorta replacement grafts are stiff with altered geometry, which can lead to downstream descending aorta distention and aneurysm (86). Presently, prophylactic surgery for aortic dilatation is guided by aortic diameter and presence of risk factors that would predispose one to aortic complications (85). With a spectrum of clinical presentations it is difficult to ascertain whether this is justified, as up to 59% of BAV aortas dissect below the surgical threshold diameter of 55 mm, while large aortic aneurysms can be stable for years (30). The underlying etiology of BAV aortopathy is likely a result of complex interplay that is beyond only hemodynamic alterations or simply a genetically predisposed aorta, thus aortic diameter alone is not a sufficient determinant of which BAV patients need surgery (71).

Numerous factors affect rate of aortic dilatation in BAV. Different studies have shown that aortic wall shear stress (WSS) is a major factor associated with ascending aorta growth (49, 81, 87). Normally, physiologic WSS decreases with age (88). In BAV, the skewed orifice jet and displaced flow results in specific patterns of elevated systolic WSS. BAV patients have abnormal right-handed helical flow that impinges on the greater curvature of the ascending aorta resulting in increased WSS (89). The jet skewness, velocity, and shear stress overloads have demonstrated to coincide with the aortic wall region prone to dilatation in a way that is specific to the cusp fusion phenotype. As previously discussed, R-L BAV exhibits increased WSS at the root and proximal ascending aorta greater curvature, and this promotes root and mid-ascending aorta dilatation that presents as a type 2 aortopathy (90, 91). In comparison, a R-N BAV velocity jet is directed toward the posterior distal aortic wall promoting arch dilatation as a type 3 or sometimes type 1 aortopathy (92, 93). This suggests a mechanistic link between BAV fusion morphology and aortopathy expression.

## Other factors contributing to bicuspid aortopathy

Elasticity and recoil are crucial functions of the aorta during systole and diastole. These functions are facilitated by the extracellular matrix (ECM) components and architecture of the medial layer of the aorta. The ascending aorta of BAV patients have been found to have altered biomechanical properties and ECM protein dysregulation due to elevated WSS forces, which contributes to the progression of aortopathy (76, 94–96). Two major structural ECM proteins are elastin and collagen, and their content is altered in BAV (97). One mouse model demonstrated that deficiencies in elastogenesis cause ECM disorganization, inflammation, and aortic valve disease (97, 98). Moreover, BAV aortas demonstrate perturbed elastin metabolism, which manifests with increased medial elastin degradation and decreased elasticity, resulting in increased stiffness (76, 99). The aneurysmal aorta of BAV patients shows increased collagen-related strength, stiffness, and alignment with overall decreased wall thickness, which contributes to its susceptibility to aneurysmal dilatation (100). The decrease in wall thickness may be directly related to collagen deposition due to elastin fragmentation. From a mechanical point of view, due to higher stiffness and strength of collagen compared to elastin, a lower degree of wall thickness is sufficient for achieving the same structural behavior. Indeed, the elastin in these areas is less abundant, less aligned, and contains greater distances between layers. This is associated with lower delamination strength and an increased risk of dissection (85). Furthermore, a recent study by Deveja et al., found that intact wall and layer-specific failure stretch and stress was significantly higher in ascending thoracic aorta aneurysm (ATAA) of BAV patients when compared to non-aneurysmal aortas and ATAA of tricuspid aortic valve patients (101). Interestingly, their findings suggest that BAV-ATAA is not associated with an increased susceptibility to dissection initiation (101). Moreover, biaxial load testing has shown that aneurysmal growth may be driven by greater elastic energy (defined as the area under the stress-strain curve) in the proximal ascending aorta of BAV patients compared to those with a tricuspid valve (102). However, a loss of actin in the inner media and increased intimal thickness due to flow-induced stress has also been found in both groups of patients, suggesting that hemodynamics may not play as large of a role in the development of aortopathy (103). Adding further complexity, in addition to WSS on the aortic wall layers, oscillatory shear stress (OSS) has also been found to be elevated in BAV (40). OSS is related to WSS as it demarcates areas where the direction of the shear stress changes multiple times in a small area. This adversely impacts the endothelium since its role is to return to homeostasis in response to non-physiologic flow. Indeed, the endothelium is healthier when subjected to laminar flow with lower OSS and mid-range WSS (104).

Moreover, WSS can also contribute to aortopathy through its impact on aortic wall stiffness. There is evidence showing that increased circumferential stiffness and elastic fiber thinning in areas of the BAV aortic wall is affected by high WSS (75). Aortic wall stiffness is also known to be associated with ATAAs and cardiovascular events and may thus be a marker of aortopathy progression (94). However, it is not yet clear whether aortic stiffness is due to a primary genetic defect or the result of a secondary adaptation to BAV flow disturbances (105). Nevertheless, both BAV and tricuspid valve ATAAs demonstrate increased aortic stiffness that does not differ between BAV and tricuspid aortic valve patients. Our group has observed that aortic stiffness may be more linear in the BAV population, possibly resulting in stiffer behavior at physiologic levels of strain and potentially less stiff than tricuspid aortic valve aortas at supra-physiologic degrees of strain (106). Nevertheless, this overlap suggests that ECM irregularities may not be specific to BAV but rather to aortic aneurysmal tissue in general (107).

Aortic mechanoreceptors detect abnormal WSS to produce different biological responses in endothelial cells and smooth muscle cells. Additionally, turbulent blood flow activates remodeling and inflammatory pathways (108). Areas of elevated WSS are also associated with increased smooth muscle cell death (109). Furthermore, premature calcification of the aortic valve is thought to be related to altered hemodynamics and over half of BAV patients older than 35 years of age will develop early onset calcific aortic valve disease that quickly progresses to aortic stenosis within 10–12 years (92). The process of calcification is thought to be related to an upregulation in pro-calcification factors caused by micromechanical forces experienced by a BAV aorta (110). Also of note, the presence of aortic stenosis or aortic insufficiency exacerbates the magnitude of WSS in BAV patients and increases the risk of dilatation (111, 112). In aortic stenosis, the enhanced WSS eventually overrides previous flow patterns associated with BAV, making BAV with severe aortic stenosis WSS patterns indistinguishable from tricuspid valves with severe aortic stenosis (113, 114).

**Figure 3:**
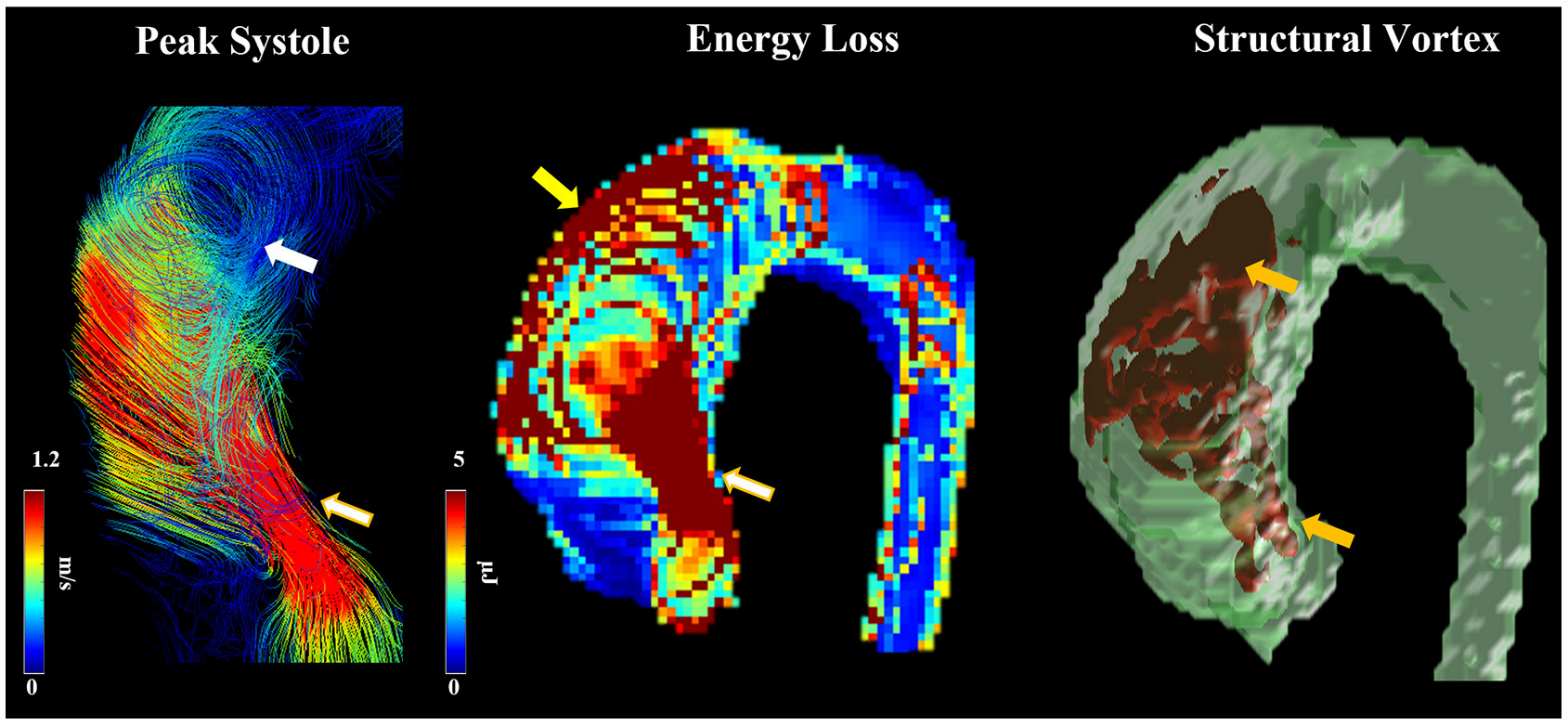
Abnormal helical flow and dilation. Flow patterns at peak systole were observed in a patient with RN fusion. Arrows point to regions with high helicity (white arrow) and vorticity (orange arrows), abnormal jet (white-golden arrows), elevated energy loss due to flow impingement (yellow arrow).

Moreover, BAV is associated with a greater total pressure gradient along the aorta distal to the aortic valve and higher viscous energy loss compared to tricuspid valves. An increase in loss of both pressure and energy are known to be related to abnormal helical flow and aortic dilatation in BAV patients (Figure 3) (74, 78). BAV also causes increased reverse flow and reduced stasis of blood (70). Finally, regions of high WSS also contain decreased endothelial nitric oxide synthase (eNOS) expression in non-dilated BAV, suggesting that alterations in eNOS expression occur independent of aberrant hemodynamics in BAV and may have a genetic etiology (115). Interestingly, proteomic analysis of BAV ATAA tissue and *NOTCH1* knockdown mice studies demonstrated that BAV aneurysmal tissue has impaired mitochondrial dynamics with attenuated fusion (116). This abnormality could be partially rescued with mitochondrial fusion activation, presenting a potential therapeutic target for BAV ATAAs (116).

## The application of 4-dimensional flow magnetic resonance imaging (4D flow MRI) in BAV

**Figure 4:**
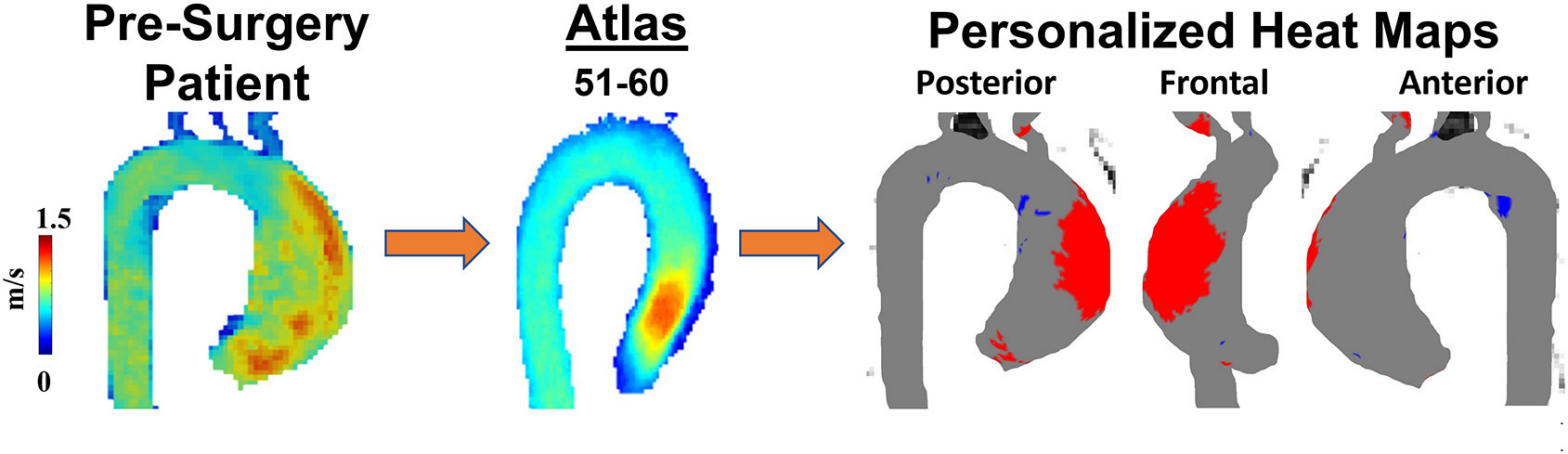
Personalized heat maps. A 60-year-old man with BAV Type 1 RL phenotype was scanned to obtain 4D flow velocities prior to surgical planning. Patient's velocity field was compared with an age and sex-match atlas allowing to identify abnormal regions (high wall shear stress in red, low wall shear stress in blue) of wall shear stress using heat maps.

Four-dimensional cardiac magnetic resonance imaging (4D flow MRI) has become a central feature for assessing and following patients with BAV and bicuspid aortopathy. Images obtained from 4D flow MRI are both quantitative and qualitative and can be used in patients with known BAV and those who have had a surgical intervention to replace their BAV (117, 118). 4D flow MRI can reveal the relation between aortic flow patterns and regional WSS (119). As magnitude of WSS correlates with pathologic remodeling and aortopathy, 4D flow MRI WSS measurements provide an important risk-stratifying clinical tool that can identify aortic regions at risk of dilatation and devastating complications (120). The first step to developing this prognostic tool is creating a reference atlas of normal physiologic WSS patterns (Figure 4) (73). Generating individualized heat maps that identify abnormal WSS patterns can guide resection strategies and improve outcomes in patients with BAV (72). Moreover, WSS may also be used to follow post-operative changes after surgical restoration of normal anatomy in BAV aortopathy. Indeed, WSS patterns have been shown to improve post-aortic valve replacement in BAV patients with aortic insufficiency (117). However, methods for quantifying WSS must be generalizable to all patients with BAV (81). Gordon and colleagues have developed a standardized and reproducible 4D flow MRI workflow with prospective application to WSS and other BAV-related hemodynamic measurements (121). Although WSS can be a promising marker of aortopathy, one study found that, at 3 years follow-up of BAV patients, aortic wall areas with high WSS had no significant anatomical remodeling (122). This suggests that although WSS alterations may precede aortopathy and contribute to its progression, this is a very slow process that probably occurs over years. Moreover, 4D flow MRI WSS has been shown to be likely underestimated due to limited temporal and spatial resolution and thus may need complementary techniques for evaluating bicuspid aortopathy (123). Such data is critical when managing patients with BAV and bicuspid aortopathy as the timing of surgical resection should be contextualized against the relative growth rate of the aneurysm.

Other alterations in aorta hemodynamics observed in BAV may be applied in conjunction with WSS to stratify risk in surgical BAV patients. Dilated R-N BAV aortas demonstrate increased flow displacement and overall flow angle at the sino-tubular junction that is associated with a wider distal ascending aorta diameter (124). Flow displacement measures the displacement of systolic flow from the centerline of the aorta and is normalized by aortic diameter (125). It can be easily derived from 2-dimensional phase contrast data to quantify aortic flow eccentricity. It is also a metric that is easier to measure than WSS (81). Dilated BAV aortas experience elevated flow skewness and eccentricity, retrograde flow, and right-handed helical flow, which may contribute to progression of aortic enlargement (126). Moreover, although aortic root flow eccentricities may not be necessarily altered in aneurysmal bicuspid aortas compared to tricuspid aortas, flow eccentricities have been shown to correlate with WSS and blood helicity, which are known to contribute to aortopathy (95). Finally, a BAV MRI pulsatile flow circulation model has been used to quantify outflow jet eccentricity (10). The model has demonstrated that asymmetric BAV outflow jets are directed at the aortic wall facing the smaller leaflet, and these regions may be at increased risk of aortic events (10). Cardiac magnetic resonance imaging can also be used to determine aortic pulse wave velocity (PWV). PWV can be used to measure aortic wall stiffness, where higher PWV has been found in BAV patients and been associated with aortic dilatation (105, 127). In contrast, Singh et al. found that PWV was not elevated in patients with bicuspid aortopathy (128). Despite these conflicting results, aortic stiffness is known to be predictive of aortic dilatation in the Marfan patient population (129). Future studies may better delineate whether aortic stiffness can be a valid predictor of aortopathy in patients with BAV. Overall, these patterns in altered hemodynamics may further compound WSS and correspond to an increased risk of aortopathy.

## Ancillary imaging options for assessing BAV biomechanics

Additional imaging strategies can be used to complement 4D flow MRI to provide a more comprehensive assessment of BAV and improve risk stratification of patients. For example, echocardiography speckle-tracking imaging identifies early abnormalities in aorta elasticity (130). Left ventricular myocardial strain analysis correlates to myocardial remodeling and may help inform decision making in BAV patients (112), while computational fluid dynamics provide more precise WSS measurements (94). In turn, WSS of the BAV leaflets may be measured by fluid structure interaction (FSI) (131). The advantage of FSI is that it provides stresses in the aortic wall (in addition to WSS), where such a calculation for WSS may be more accurate because the wall is modeled as elastic and not simply as a rigid boundary. FSI also provides measurements of vessel structural stress, which correlates with aortic media degeneration (132). Machine learning has also been employed to classify BAV aortas at risk of dilatation using 4D flow MRI parameters (133). Machine learning uses computer systems that can “learn” from datasets by using statistical models and algorithms to identify patterns in the data. Such a strategy may facilitate more accurate and efficient predictive models and streamline prognostication for BAV patients. It is important to emphasize that a complete imaging-based assessment of the heart and aorta should be performed for patients with BAV. Valve function may be assessed in detail using echocardiography and cardiac MR (56), whereas 4D flow MRI and CT scans can be done to generate accurate and informative images of the aorta in BAV patients (134).

These studies collectively confirm the safety, feasibility, and potential clinical applicability of 4D flow MRI in assessing and following BAV patients. They also show some of the limitations associated with this imaging modality, which are amplified by the heterogenous nature of the BAV patient population. Future studies should include larger number of healthy controls and BAV patients to map normal physiological hemodynamics and identify BAV patients at risk of deadly aortic complications. Indeed, a personalized medicine approach to BAV aortopathy clinical decision-making involves detecting flow derangements with 4D flow MRI and other imaging modalities; assessing biomechanical wall properties; measuring the levels of circulating biomarkers of wall remodeling and endothelial dysfunction; and identifying genetic alterations. Such parameters can be used to help determine which, if any, regions of the aorta need to be resected; guide in the timing and extent of any potential surgical interventions; and provide prognostic insight into the clinical course of aortopathy.

## Conclusion

Bicuspid aortic valve is a congenital heart disease that affects up to 2% of the general population. In addition to placing those patients at a higher risk for valvular dysfunction, BAV predisposes patients to developing bicuspid aortopathy. While some factors have been identified, our knowledge of the potential genetic causes for BAV and bicuspid aortopathy remains incomplete. Moreover, our understanding of the altered hemodynamics and biomechanics that preside in this patient population continues to evolve. Such an understanding has been augmented by novel, non-invasive multimodality imaging techniques. Collectively, these advances have been translated to clinical practice, where care providers and surgeons are now able to base their management plans on more accurate evidence. This is an exciting area that seamlessly blends principles in biomedical engineering with advanced imaging techniques and precise treatment strategies that can be personalized. Mathematical modeling, machine learning, and other non-invasive biomarkers can herald the next generation of impactful innovations for patients with BAV and bicuspid aortopathy.

## Author contributions

AF and JG: contributed to conception and design of this paper, acquisition and interpretation of information, drafting and critically revising manuscript for intellectual content, and has approved the final version of manuscript. MK, ED, and PF: contributed to drafting and critically revising manuscript for intellectual content and has approved the final version of manuscript. All authors contributed to the article and approved the submitted version.

## Funding

JG received funding from the University of Calgary URGC SEM #1054341 and start-up funds. The Natural Sciences and Engineering Research Council of Canada/Conseil de recherche en sciences naturelles et en génie du Canada RGPIN-2020-04549 and DGECR-2020-00204.

## Conflict of interest

The authors declare that the research was conducted in the absence of any commercial or financial relationships that could be construed as a potential conflict of interest.

## Publisher's note

All claims expressed in this article are solely those of the authors and do not necessarily represent those of their affiliated organizations, or those of the publisher, the editors and the reviewers. Any product that may be evaluated in this article, or claim that may be made by its manufacturer, is not guaranteed or endorsed by the publisher.
